# Learning in the zone: toward workforce development of evidence-based public policy communication

**DOI:** 10.1186/s12889-018-5617-0

**Published:** 2018-06-05

**Authors:** Beth E. Meyerson, Laura T. Haderxhanaj, Karen Comer, Gregory D. Zimet

**Affiliations:** 1 Rural Center for AIDS/STD Prevention, Indiana University School of Public Health-Bloomington, Bloomington, Indiana USA; 2 Department of Applied Health Science, Indiana University School of Public Health-Bloomington, Bloomington, Indiana USA; 3 Center for HPV Research, Indiana University School of Medicine, Indianapolis, Indiana USA; 4 The Polis Center, Indiana University Purdue University at Indianapolis, Indianapolis, Indiana USA; 5 Department of Adolescent Medicine, Center for HPV Research, Indiana University School of Medicine, Indianapolis, Indiana USA

**Keywords:** Health policy communication, Public health workforce development, Human papillomavirus, State policy development

## Abstract

**Background:**

Evidence-based policy communication (EBPC) is an important, emerging focus in public health research. However, we have yet to understand public health workforce ability to develop and/or use it. The study objective was to characterize capacity to develop and use EBPC and identify cooperative learning and development opportunities using the case of Human papillomavirus (HPV).

**Methods:**

Vygotsky’s Zone of Proximal Development (ZPD) informed guided interviews with 27 advocates in Indiana from government, industry, research, state associations and individuals. Participants focused on HPV, cancer, women’s health, school health and minority health.

**Results:**

Capacity to develop and use EBPC was reported to develop through cooperative learning opportunities on the job or in advocacy focused coalitions. Coalition learning appeared to translate across health topics. Notably, policy experience did not assure understanding or use of EBPC.

**Conclusions:**

The ZPD framework can inform workforce EBPC interventions by focusing on actual development, potential development and factors for learning and development in the ZPD. Future studies should further clarify and evaluate emerging indicators in additional public health policy areas with a larger sample.

## Background

Evidence-based policy communication (EBPC) is relatively new and is based on Brownson et al.’s conception of evidence-based policy: conveying evidence-based public health interventions to policymakers [1–4]. EBPC is an important, emerging part of public health research in response to calls for empirically-based efforts to translate public health evidence for use in the policy arena [5, 6].

Successful EBPC has the potential to transform the health landscape by influencing the policy decision-making process. However, before such success can be achieved, the ability (or inability) of the public health workforce to develop and use EBPC to advance policy goals must be understood and deficits addressed. We know there is a substantial gap between public health workforce expertise in specific areas and the ability to translate that expertise into the policy domain, because policy development remains the weakest of public health’s core functions [7–9]. This gap persists even with wide acknowledgment that structural conditions, largely determined by policy, powerfully impact population health [10–12]. As Brownson and colleagues noted, public health training programs do not sufficiently focus on policy knowledge and skills development; [1] though this will likely change as accredited schools of public health are required to offer policy learning opportunities to all students [13]. While this is good news, gains will be realized only in the future, and depend upon the quality of that education. Because the field of public health is not comprised only of those with public health degrees, [14] efforts to develop EBPC capacity in the public health workforce must be bifocal: occurring through formal public health education programs and in the field. Understanding workforce capacity for EBPC, then, is a first step.

A few studies have attempted to understand public health workforce policy capacity by examining policy behaviors. Examples of these behaviors are summarized in Table 1, though it is important to note that a few citations are editorials and not studies. They are, however, important contributions that point the way toward development of measurable policy behaviors.

**Table 1:** Examples of policy behaviors from the literature, 2016

Behavior	Citation
Prepare issue briefs for policy makers	Harris and Mueller, 2013 [16] Meyerson and Sayegh, 2016 [15]
Publish a state policy agenda	Meyerson et al., 2003 [17]
Publish consensus or other evidence-based document aimed at policy change	Friedlaender and Winston, 2004 [18]
Advance model public health legislation, regulation or ordinance	Hartsfield et al., 2007 [25]
Publish policy implications as part of research publications	Giles-Corti et al., 2015 [26]
Give public testimony to policy makers	Harris and Mueller, 2013 [16] Meyerson et al., 2003 [17] Meyerson and Sayegh, 2016 [15]
Communicate with legislators, regulatory officials, or other policy makers regarding proposed regulations, legislation or ordinances	Harris and Mueller, 2013 [16] Meyerson and Sayegh, 2016 [15]
Provide technical assistance to a legislative, regulatory or advisory group for drafting proposed legislation, regulation or ordinance	Harris and Mueller, 2013 [16] Meyerson and Sayegh, 2016 [15]
Program disseminates STD related information to policy makers (e.g. epidemiologic reports)	Meyerson et al., 2003 [17]
Participate on a board or panel responsible for health policy	Harris and Mueller, 2013 [16] Meyerson and Sayegh, 2016 [15]
Program works with a state coalition on STD related issues	Meyerson et al., 2003 [17]
Staff contact policy makers as individual citizens	Meyerson et al., 2003 [17]
Conduct policy surveillance	Brownson et al., 2009 [1]
Conduct media advocacy	Chapman ad Lupton, 1994 [27] Wallack et al., 1993 [28]

The Meyerson [15] and Harris [16] studies used five policy behaviors measured by the National Association of County & City Health Officials in periodic surveys of U.S. local health departments. Meyerson et al.’s 2003 study measured similar behaviors among state sexually transmitted disease (STD) programs [17]. These local health department and STD program studies were limited by the unit of measure: one person reporting program or leadership behavior, and period of measure: they were snapshots in time and therefore do not speak to potential for future development. Further, while these indicators are examples of policy behaviors, they are not necessarily evidence-based; which, according to Brownson, means that they express information grounded in both quantitative (e.g. scientific findings) and qualitative (e.g. narrative accounts) evidence, [1] and based on an awareness of the policy audience [2, 18].

**Fig. 1:**
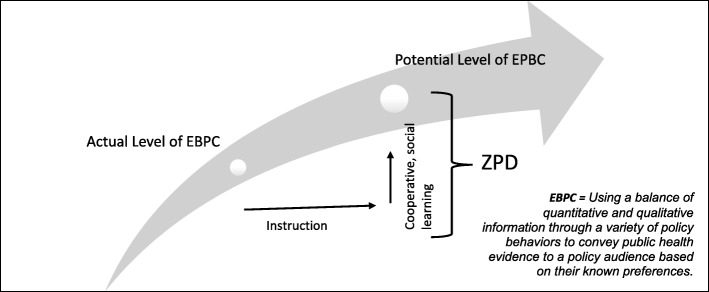
Zone of Proximal Development (adapted) for Evidence-Based Policy Behaviors (EBPC). Meyerson et al., 2016

A useful framework to help clarify and address the central challenge of EBPC development over time is Vygotsky’s theory of the Zone of Proximal Development (ZPD). This learning/development theory presupposes a dynamic and socially-oriented relationship between learning and development [19]. Briefly put, ZPD theory seeks to define the upper boundary of the realm of abilities a person already possesses and the lower boundary of abilities beyond that person’s capacity at the time. The gap between these two boundaries defines the ZPD, that is, the area where a person can learn new goals or, in this case, evidence-based policy behaviors with guidance. Vygotsky held that effective teaching targets the ZPD, with the teacher helping learners bootstrap their way dynamically to acquire greater skills and knowledge. In contrast, ineffective teaching aims too high (leading to frustration and a lack of development) or too low (leading to disengagement and lack of development). Identification of the ZPD essentially entails a defining of the *potential level of development* in order to understand what maximally could be accomplished through problem solving experience or guidance in social learning contexts [20]. Using ZPD theory, we argue that advancing public health workforce EBPC skills requires more than an assessment of the *current level of (EBPC) development and knowledge*, which simply provides a starting point for intervention development. When applied to public health workforce EBPC skills, the ZPD can help clarify types of and platforms for workforce interventions (see Fig. 1). Further clarity about optimum policy behaviors and capacities will ultimately be necessary to fully articulate EBPC behavior and capacity development expectations. For now, however, Brownson’s definition of using a balance of peer reviewed quantitative and qualitative evidence through a variety of behaviors (Table 1) based on audience preference is a good start.

Using ZPD, the research task includes describing and characterizing current public health policy knowledge and behaviors (actual level), clarifying optimum EBPC behaviors (potential level), and identifying situational or structural opportunities for learning and development within the ZPD.

To explore the application of ZPD to EBPC skills and development, an exemplar issue was chosen which is frequently challenging in U.S. policy contexts: human papillomavirus (HPV). Specific study aims included the conceptualization of policy and EBPC to advance HPV and cancer-related outcomes, identification of actual and potential development levels for HPV EBPC, and identification of potential ZPD elements.

## Methods

In-person, 60-min interviews were conducted with individuals engaged in HPV, cancer and public health policy work in Indiana from October–December 2015. We focused on the state policy process because of its importance to public health policy [21].

The interview guide contained 12 open-ended questions exploring conceptualization of policy, EBPC, reported capacity to develop and use EBPC, and reactions to a ‘mock’ policy brief (“mock-up”). The mock-up was presented in the third portion of the interview and written with anonymized text using a Lorem ipsum generator to allow the observation of participant reaction and developmental thinking when presented with a partial presentation of EBPC for HPV. Interviews were recorded and transcribed for analysis with Nvivo software (QSR International, v.9).

Purposeful sampling drew one individual from each of the following types of organizations: state associations, community coalitions, clinical care research, local and state government agencies, state legislature, and individual advocates. All participants were previously observed by authors as involved in HPV or cancer policy communication.

Indicators of current knowledge and behavior measured participant level of actual development for EBPC. Reported current or prior exposure to or participation in collaborative policy learning environments, and response to the mock-up characterized the ZPD. Ratings of “Low, Moderate, and High” were assigned for the potential to develop and use EBPC for HPV. Ratings were assigned based on researcher evaluation of knowledge accuracy, the congruence between policy goals and audience, and the reporting of concrete examples of EBPC develop and/or use. Table 2 displays the application of ZPD theory with associated study measures and explanations.

**Table 2:** Evidence-Based Policy Communication (EBPC) Study indicators by Zone of Proximal Development (ZPD) Components, 2016^a^

Level of actual development	Zone of proximal development	Level of potential development
Accurate knowledge about policy *What policy is and where it happens*	Exposure to or participation in collaborative policy learning *Experience with policy learning on the job, in association context, other*	Rankings for potential to develop EBPC for HPV *Researcher assigned: Low, Moderate, High*
Accurate knowledge about EBPC (what it is) *EBPC: Using a balance of quantitative and qualitative information through a variety of policy behaviors to convey public health evidence to a policy audience based on their known preferences.*	Exposure to EBPC examples from others *Reported exposure through peers, peer organizations, on the job, other*	Rankings for potential to use EBPC for HPV *Researcher assigned as Low, Moderate, High*
Accurate knowledge about goals for policy communication and audience *Policy communication informs decisions about administrative or legislative policy. Awareness of audience preferences for communication (how much narrative, how much data, what type of data)*.	Prior/current experience in environments which have potential to enhance social learning about EBPC *Reported experience (current or past)*	
Reported policy behavior indicating understanding and use of EBPC *Reported examples of past or current EBPC.*	Proximity to the policy process *Working in state policy process full time, part time, sometimes, seldom within the last 3–5 years.*	
	Response to EBPC “mock-up” *Recognition of this type of communication, impact of mock up on interview discourse, engaged discussion (participant driven) about EBPC based on mock up.*	

^a^Explanatory text in italics

Coding was a priori, based on Table 2 indicators, and also open to allow further study exploration. Two researchers independently coded data and met to compare and resolve conflicts after reviewing a sample of interviews (*N* = 10). Final coding was then conducted using the agreed scheme with the entire group of interviews. Coded data were then classified as low, moderate and high for each of the learning/development levels. Results are presented as theme-related tables and as interview quotations for deeper understanding. The study was deemed exempt by Indiana University Institutional Review Board.

## Results

The sample consisted of 27 individuals across various public health and policy organizations. State association participants (25.9%, 7) included associations of school health nurses, rural health providers, local health departments, action agencies focused generally on health and human services, and organizations focused on cancer, HPV or reproductive health. Community level organizations (22%, 6) included those focused specifically on minority health, health care coverage, reproductive health services, cancer, and immunizations. Clinical care and research participants (22%, 6) were focused on cancer or HPV specifically. Local and state government participants (14.8%, 4) included public health departments, state legislators and state programs focused on cancer. Industry participants (7.4%, 2) were focused on HPV immunization, testing or cancer treatment. Individual advocates (7.4% 2) operated in the policy process focusing on HPV vaccination, screening and/or cervical cancer treatment. About one third of participants (33.3%, 9) focused specifically on HPV or cervical cancer, 25.9% (7) on immunizations, and 33.3% (9) on cervical screening; and 40.7% (11) had jobs working directly in the policy process.

### Level of Actual Development for EBPC

The level of actual development for EBPC was indicated by reported knowledge and behaviors in the policy process. Most participants misunderstood public policy, policy audiences and what constituted policy behaviors. Job-related experience in the policy process appeared to correspond with the level of actual policy development. However, while participants with policy related jobs generally expressed correct conceptualizations of policy and policy behaviors, they did not necessarily know what constituted EBPC (Table 3).

**Table 3:** Indicators and Levels of Actual Development for Evidence Based Policy Communication (EBPC) about Human Papillomavirus (HPV), 2016 (*N* = 27)

Indicators	Levels of actual development with reported examples from interviews		
	High level	Moderate level	Low level
Policy knowledge	• Legislative decisions (HPV vaccination, Medicaid expansion, reproductive health services access) • Administrative decisions (Medicaid, VFC^a^)	• School principal decisions about public information in school • Private insurer decision to require HPV testing	• Public, parent or physician decision to vaccinate or access cervical cancer screening
Goals of policy communication	• Remove barriers to access • Invest in programming • Require standard of care vaccination or screening	• Support public discussion (school) about HPV, vaccination and/or screening • Reimburse for screening (private insurers)	• Increase knowledge of HPV and related cancer • Change opinion about benefit of screening or vaccination among parents, teachers, providers
Evidence	• Data from peer reviewed studies • Economic data • Epidemiologic data • Stories from those affected	• Stories from those affected (Only and not with other evidence) • Some studies or information from CDC about HPV or cancer^b^	• Information about coalition messenger(s) • Any information about HPV or cervical cancer
EBPC knowledge	• Integrating evidence with desired policy change for specific policy audience	• Education about HPV and/or cervical cancer primarily for non-policy audience (what it is)	• Not sure what it is, what it looks like, or how to use
Reported EBPC behaviors	Use of: • Policy report cards • Policy briefs • Grassroots coaching for letters, testimony, media contact, meetings	• Public information about HPV and cervical cancer • Screening and vaccination recommendations for doctors or insurers	• Giving testimony to legislature (The act but without quantitative or qualitative evidence)

^a^*VFC* Vaccines for Children Program

^b^*CDC* Centers for Disease Control and Prevention

As shown in Table 3, participants with low or moderate knowledge of policy tended to confuse policy with HPV information, hospital policy, or parent, physician or private payor behavior. For these participants, policy was construed as education for these audiences to encourage them to vaccinate/screen or to underwrite services. Those demonstrating correct knowledge about policy primarily conceptualized it as state legislation, and only four participants discussed policy as administrative action (funding, regulation, et cetera) by state agencies or the governor. A correct example of administrative policy was given by this participant:

*Medicaid made a change to their policy to cover all adults in pharmacies….But what they did at the same time was* (to require) *all Medicaid eligible VFC children to get their vaccines from a VFC provider, which meant basically that the pharmacy could no longer bill for it.*

Those participants confusing HPV or cervical cancer education with policy notably did not discuss policies focused on education such as state vaccine laws mandating education of parents or providers. While some participants believed that increased knowledge about HPV or cervical cancer would generally lead to “better HPV policy,” the concept of “HPV policy” was never disentangled from information about HPV or cervical cancer. Those with correct policy knowledge discussed policy behaviors akin to those listed in Table 1 such as advocacy or policy maker education, policy monitoring and the production of policy communications for specific audiences.

*We engage in the full array of policy advocacy from monitoring bills to doing educational forums and we educate members to actively lobby, both at the grassroots level and direct lobbying of legislatures and administrators at the state and federal level.*

The confusion of policy and education appeared to be connected with an understanding that EBPC meant talking only about cervical cancer or HPV epidemiologic evidence such as diagnosis or death rates, or information about the virus itself. There were, however, correct examples of EBPC tools informed by such evidence and developed to achieve policy goals. These included policy briefs or ‘report cards’ focused on a specific policy outcome, the use of evidence in legislative testimony, or evidence-based policy recommendations.

*We have an annual report* (which is) *our ultimate policy brief ranking states in 11 different policy areas. It provides the background of what the problem is, a policy solution, and an issue overview that includes a lot of evidence in statistics about why that issue is important.*

Despite the reported policy awareness of some participants, most of the sample did not appear to understand EBPC. Only those that developed “policy briefs” reported concepts about what EBPC might involve beyond providing written information specifically about cervical cancer or HPV as health issues. Those with stronger EBPC knowledge tended to have work-related experience in the policy process and had some capacity to develop EBPC. That said, experience working with the legislature was not sufficient to develop participant knowledge or capacity to use EBPC. For example, one participant with years of legislative experience stated that the evidence informing any policy communication was about the coalition members themselves.

*We try to develop a plan of what we can say* (to legislators) *in a short amount of time, not more than one page, probably double spaced so that we can say one, two, three, this is what we see, this is the facts we know, this is what we would like to see happen and this is why we’d like to see it happen. However, there is evidence, which is that our members* (as a profession) *are trusted.….I have to say I don’t think I’ve taken any* (data)*.*

What constituted public health policy evidence ranged for participants. For those who functioned in the policy process, several indicated that a balance was needed between research or studies about the effectiveness of screening and follow-up investments and personal narratives. Participants felt this was particularly the case for HPV, given the tremendous social judgement about sexual activity. To navigate moral policy, one participant stated that “[Something] *I’ve actually learned over the years is that I sometimes am more powerful if I give anecdotes* [rather] *than evidence.”*

Challenges identifying and presenting evidence were reported by several participants at all levels of development. Some participants felt the burden of *“wading through CDC data”* to identify the latest information about HPV when preparing to talk with state lawmakers. Others noted the challenge of synthesizing the most recent studies of HPV and cervical cancer, and translating the information into understandable concepts. Those rated as having high levels of actual policy development were challenged to find policy related evidence, such as the impact of vaccine policy, or public funding for alternative screening venues.

### Zone of Proximal Development

Participants discussed how they obtained their policy knowledge, learned policy behaviors, and learned about EBPC. Every example was social and work-related instead of formal education-based; and each example included collaborative learning or policy modeling.

(I learned) *on the fly. You really learn how to put together a good policy brief by trial and error, and through that communications process of giving that information to your various audiences and them asking questions or telling you that they need more information.*

Over half of participants reported experience with policy coalition work – whether led by the participant’s organization, or whether the participant was part of a larger coalition focused on some aspect of health policy. These participants spoke about coalition experience as contributing to their policy learning. Notably, coalitions discussed by participants were not necessarily focused on HPV or cervical cancer, as less than half reported being part of such a coalition. However, the cooperative policy learning environment from any policy-related coalition experience appeared to be transferrable from issue to issue and endured over time.

*Okay, here’s how I learned to* [put together a good policy brief]. *Being part of the HIV Community Planning Group and at some sort of public forum, only having like a minute or less to talk about HIV, and trying to get a whole lot of important stuff into this minute or less.*

One participant reported active engagement in the development of EBPC communication skill and knowledge with the organization’s coalition members in order to, in this example, further healthcare funding policy:

*So I really say to* (the coalition members): *the first thing you need to have with you is data, so let’s talk about the number of uninsured in your county. Maybe 2013 is a good benchmark ‘cause that was pre-ACA, and then write down how many enrollments happened in 2013 and 2014. The numbers really speak for themselves.*

While participants ranged in EBPC knowledge and practice, everyone had a response to the EBPC mock-up. These responses ranged from conceptualizations of it as “*another way to organize the information*,” to active and evolving reflection about issues related to the gathering and framing of evidence for policy argument. Participants spoke of capacity (or lack of) to develop similar material and the usefulness of it; especially with a moral policy issue such as HPV. Here, the challenge was one of balancing evidence about policy effectiveness such as HPV vaccine policy with stories about human impact.

### Level of Potential Development

Levels of potential development began to arise after considering the actual level of development and emerging indicators of the ZPD. We classified potential development levels based on participant identification of zone indicators. For example, over half of the sample (60%) was categorized as moderate to high potential to develop and or use HPV EBPC because they reported opportunity for learning and development through modeling, guided example in job or coalition environments (see Table 4).

**Table 4:** Zone of Proximal Development (ZPD) Indicators for Evidence-Based Policy Communication (EBPC) about Human Papillomavirus (HPV) by Levels of Potential Development, 2016

ZPD Indicators	High level	Moderate level	Low level
Exposure to or participation in collaborative policy learning	• Is an advocacy coalition member • Has a policy role in organization	• Has coalition experience, but no recognized collaborative learning	• Has no exposure. Receives no coaching from others.
Exposure to EBPC of others	• Has developed or co-developed EBPC	• Has used, but not developed • Has observed others using	• Has no exposure.
Prior/current experience in environments with potential for social learning about EBPC	• Is a policy coalition member • Has a work environment with potential for EBPC learning	• Could join a policy coalition with EBPC potential. (Awareness of such a coalition) • Has potential to work with others through job (but not currently)	• Is a policy coalition member (though unlikely that coalition has potential to develop or use EBPC)
Proximity to the policy process	• Has full time policy job • Has a policy role for organization but not primary job	• Has periodic policy process engagement	• Gave policy testimony once or twice before
Response to Mock-up	• Has clear recognition of EBPC tool(s) based on mock-up • Demonstrated advanced thinking and conversation about EBPC and how tool might be used	• Demonstrated moderate recognition of EBPC tool after exposure to mock-up • Unclear how to use it	• None to slight recognition of mock-up as an example of EBPC. Focused more on format than concept. • Unclear whether could or would use

Those rated as having moderate and high EBPC potential tended to have a job that required full-time participation in the policy process. An exception to this involved- two participants who held jobs in organizations that limited their engagement with the policy process; however, their role was to prepare coalition members for it. Participants reporting one or less than one engagement in the policy process since 2013 were rated as low for the capacity to develop and/or use HPV EBPC because of the limited opportunity to learn and practice it. Further, while coalition engagement appeared to be a policy learning ground for many participants, being in a cancer-related or HPV coalition did not necessarily translate to a high policy development classification especially if participants did not report collaborative policy learning or development opportunities.

## Discussion

This exploratory study suggests that EBPC development interventions for the public health workforce would benefit by using the ZPD framework, because the ZPD helps to elucidate current activity (actual), facilitators of knowledge and skills development (zone), and estimates potential for future skills and knowledge. The ZPD framework also embraces the permanently iterative relationship between learning and development. This is good in the long run, because if one were to solely measure actual policy knowledge and skill levels and presume that those rated high for actual development were in no need for further development, we would miss an important observation from this study: that those who were active in the policy process still did not necessarily understand, develop or use EBPC.

Discussion emerging from encounters with the mock-up provided a good opportunity to observe potential for future development and knowledge; however, the estimates for potential development were limited because they are unverifiable without a retrospective study design relying on participant self-report of historical learning and development, or a prospective design over a longer period of time with focus on measuring knowledge and observing behaviors and EBPC tools used. We recommend both retrospective and prospective study designs for this purpose.

The emergence of self-reported zone indicators such as job focus, policy proximity, and coalition experience provide a starting point for further indicator characterization and evaluation. This is because, to our knowledge, there is no theory of policy learning and skills development beyond the focus on the evolution of policy ideas [22, 23]. As ZPD indicators are further developed, it will be important to verify their precise contribution to development and learning. Their self-reported existence alone does not mean that EBPC related learning and development occurs or is even related to them. That said, participant meaning making about their own policy learning journeys should not be devalued in the absence of studies about the association between reported zone indicators and observed EBPC development.

Similarly, there remains the challenge of knowing when one encounters a correct reported example of EBPC versus something incorrectly construed as EBPC. This is a study limitation, as we did not see examples of reported EBPC in the course of the study. All participants were asked to provide examples after their interviews, but only those who were coincidentally assessed as developing and using EBPC actually did. Thus, there was no opportunity for content analysis verifying reported EBPC. Participant response to the mock-up did facilitate observation of completely independent thinking about what an EBPC tool might look like and issues with it; as if the opportunity to reflect on the mock-up was in and of itself a social learning opportunity for EBPC. That said, the vagueness (Lorem ipsum wording) might be considered a study weakness albeit developed to avoid confounding.

Our observation that several participants confused policy with education for individual level behavior change reflects findings from our prior study of state comprehensive cancer plans. In the case of the cancer plans study, this was likely because there were few policy related partners involved in the development of state plans [24]. Interestingly, in this EBPC study there was policy experience, and yet that did not necessarily mitigate the confusion of education about HPV/cervical cancer and policy.

A remaining challenge is identifying what constitutes evidence for policy communication. While a few participants reflected Brownson’s finding that evidence is a balance of quantitative and narrative data, several participants felt that evidence was only about HPV or cervical cancer as virus and condition. Only one participant identified the challenge of not having a body of evidence about HPV and cervical cancer policy, such as the impact of vaccine funding regimes, state policy incentives for HPV vaccination, cervical screening and follow-up in alternative settings, or related structural incentives such as insurance requirements to achieve vaccine and screening outcomes. The challenge is for all public health policy researchers to contribute to a body of evidence for EBPC.

## Conclusion

While the ZPD was useful to understand complexities of capacity to develop and use EBPC, future steps must be taken for more robust research. First, emerging indicators of the ZPD should be further clarified and verified. We think this is best done through interview with those who know about and engage in EBPC. Second, sampling should include a variety of public health policy issue areas to test whether the elements are shared widely or are disease/condition specific. Finally, the next iteration of studies should explore whether adaptation of EBPC behaviors is acceptable and under what conditions. These findings would assist the selection of a dissemination and implementation framework to guide policy capacity intervention design and testing.

## Abbreviations

EBPC: Evidence based policy communication
HPV: Human papillomavirus
ZPD: Zone of Proximal Development
